# Comparison of Long Non-Coding RNA Expression Profiles of Cattle and Buffalo Differing in Muscle Characteristics

**DOI:** 10.3389/fgene.2020.00098

**Published:** 2020-02-26

**Authors:** Hui Li, Kongwei Huang, Pengcheng Wang, Tong Feng, Deshun Shi, Kuiqing Cui, Chan Luo, Laiba Shafique, Qian Qian, Jue Ruan, Qingyou Liu

**Affiliations:** ^1^ State Key Laboratory for Conservation and Utilization of Subtropical Agro-Bioresources, Guangxi University, Nanning, China; ^2^ Agricultural Genomics Institute at Shenzhen, Chinese Academy of Agricultural Sciences, Shenzhen, China

**Keywords:** buffalo, cattle, RNA-seq, lncRNA, myogenesis

## Abstract

Buffalo meat consist good qualitative characteristics as it contains “thined tender” which is favorable for cardavascular system. However, the regulatory mechanisms of long non-coding RNA (lncRNA), differences in meat quality are not well known. The chemical-physical parameters revealed the muscle quality of buffalo that can be equivalent of cattle, but there are significant differences in shearing force and muscle fiber structure. Then, we examined lncRNA expression profiles of buffalo and cattle skeletal muscle that provide first insights into their potential roles in buffalo myogenesis. Here, we profiled the expression of lncRNA in cattle and buffalo skeletal muscle tissues, and 16,236 lncRNA candidates were detected with 865 up-regulated lncRNAs and 1,296 down-regulated lncRNAs when comparing buffalo to cattle muscle tissue. We constructed coexpression and ceRNA networks, and found lncRNA MSTRG.48330.7, MSTRG.30030.4, and MSTRG.203788.46 could be as competitive endogenous RNA (ceRNA) containing potential binding sites for miR-1/206 and miR-133a. Tissue expression analysis showed that MSTRG.48330.7, MSTRG.30030.4, and MSTRG.203788.46 were highly and specifically expressed in muscle tissue. Present study may be used as a reference tool for starting point investigations into the roles played by several of those lncRNAs during buffalo myogenesis.

## Introduction

As a form of striated muscle tissue, skeletal muscle is an important object in the study of meat quality and plays key roles in regulating homeostasis and metabolism, accounting for 40–60% of the animal body (Li et al., 2018b). Skeletal muscle developmental inability *via* perantes lead to embryonic death, while the failure to repair or maintain skeletal muscle after birth that can lead to a decline in quality of life and even death. Myogenic progenitor cells from multi-potent mesodermal precursor cells are committed to the muscle fate, and express Pax3 and Pax7 destined to become myoblasts (Buckingham and Relaix, 2015). Then, myoblasts experience proliferation, differentiation and fuse into myotubes through the regulation of myogenic regulatory factors: MyoD, MRF4, Myf5, and myogenin play key roles in the process of regeneration in adult muscle (Hernandez-Hernandez et al., 2017). In fact, various non-coding RNAs (ncRNAs) have been identified and demonstrated to regulate myogenesis and muscle regeneration including microRNAs (miRNAs) and long non-coding RNAs (lncRNAs) (Li et al., 2018b). MiRNAs profoundly influenced the physiology and pathology of skeletal muscle, such as miR-1 and miR-133 *in vitro* and *in vivo* have distinct roles in regulating skeletal muscle proliferation and differentiation (Li et al., 2018a).

LncRNAs have played versatile roles in regulating skeletal myogenesis and regeneration at multiple levels (Kallen et al., 2013; McHugh et al., 2015; Munschauer et al., 2018; Neumann et al., 2018). LncRNAs are well known for their involvement in transcriptional/epigenetic regulation on chromatins through interacting with chromatin regulators, for example, acting as “molecular scaffold” or decoys to activate or repress transcription (Caretti et al., 2006; Korostowski et al., 2012). Other unique mechanisms have been found to explain the diverse modes of lncRNA action in myogenesis (Han et al., 2014; Zhou et al., 2015). For example, lncRNA could act as competing endogenous RNAs (ceRNAs) to sequester miRNAs from their target mRNAs (Cesana et al., 2011; Kallen et al., 2013). Some transcribed from antisense strand of protein coding genes could directly pair with the mRNA to modulate coding gene translation (Wang et al., 2013; Wang et al., 2016). Interestingly, Emerging research has shown that some lncRNAs could translate micropeptides (<100 amino acids) to perform micropeptide-mediated functions (Anderson et al., 2015; Nelson et al., 2016). However, participation of miRNA and lncRNA in muscle development are still in their infancy, especially in livestock, for example, in buffalo studies.

Buffaloes usually used for labor purposes, but now optimized for meat or milk production (Pisano et al., 2016; Low et al., 2019). In developing countries, buffalo meat usually from old (>10 years old) period is eaten and therefore seemed tougher than beef (Sakaridis et al., 2013; Zhang et al., 2016). Compared to beef, buffalo meat indeed has less fat, lower calories, and less cholesterol, which is healthier and can confer significant cardiovascular benefits. “Buffalo meat is an amazing cure for diabetes” as is described in the Compendium of Materia Medica, which was considered to be the most comprehensive and complete medical work in the history of Chinese medicine. Here, the longissimus dorsi muscles of swamp buffaloes and Guangxi native cattle under the same feeding and management were selected, and analyzed its differences in physiological biochemical indexes. The histological staining and analytical chemistry methods were used to directly compare the differences in pH, water content, shear force, intramuscular fat content, ash content, and myofiber structure.

In the second part, the Ribo-Free RNA-seq method was selected to analyze the expression of lncRNA in longissimus dorsi muscles of swamp buffaloes and Guangxi native cattle. Differentially expressed genes and lncRNAs were identified in skeletal muscle samples, and the candidate lncRNAs were verified by Quantitative PCR(qPCR). We further constructed coexpression and ceRNA networks to select candidate lncRNA. Our research will be beneficial for the improvement of Chinese meat buffalo breeding and provide new insights into the genetic mechanism of Chinese swamp meat.

## Materials and Methods

### Sample Preparation

Chinese swamp buffalo (n = 3, 12 months old) and Guangxi native cattle (n = 3, 12 months old) under the same feeding and management were obtained from SIYE husbandry of Guangxi, China. The longissimus dorsalis muscle of adult buffalo and cattle were used for muscle quality analysis, transcriptome sequencing and qPCR analysis. The 4 months old buffalo and cattle fetal with a body length of 15 cm were selected from the local slaughterhouse in Nanning. Buffalo and cattle fetal tissues (skin, heart, liver, spleen, lung, kidney, small intestine, leg muscle, and longissimus dorsalis muscle) were used to extract RNA and analyze the expression of lncRNAs.

### Meat Quality Evaluation

The longissimus thoracis muscles were taken between the 12th and 14th ribs from the left side of body, and performed the following analyses in triplicate: the pH was immediately measured using a pH meter (Thermo Orion, Hudson, NH, USA); water content was determined on drying at 100°C for 24 h; crude fat level was assessed by extracting for 12 h using petroleum ether; ash content was evaluated by ashing at 600°C for 10 h; shear force was measured using a C-LM3 digital display tenderness instrument (Northeast Agricultural University, Harbin, China). The amino acid composition in muscle samples was determined using an amino acid analyzer. Differences between the two groups were compared using a *post hoc* test.

### Library Preparation

Total RNA of longissimus muscle was extracted and assessed by electrophoresis and quantified by NanoDrop spectrophotometer (NanoDrop, Wlinington, USA). Ribosomal RNA was removed by probe, and then the remaining RNA was used for library construction and sequencing (Ribo-Zero RNA-seq). cDNA library preparation and Illumina sequencing analysis were perofrmed as previous studies (Hui et al., 2018).

### lncRNAs Identification

Potential lncRNAs were filtered through the following highly stringent criterion: (1) transcript length is not less than 200 nt; (2) transcript expression is more than 3 reads; (3) the transcripts were annotated as “i”, “j”, “o”, “u”, and “x” according to the cuffcompare classes; (4) the coding potential calculator (CPC) score less than -1, and coding-non-coding-index (CNCI) score less than 0 were kept; (5) the transcripts containing open reading frame (ORF) is greater than 100 aa were removed; (6) *via* aligning to the Swiss-Protein, Cpat, and Pfam database, the transcripts were removed with known protein-coding domain.

### Gene Ontology and KEGG Pathway Analysis

Gene Ontology (http://www.geneontology.org) and KEGG pathway (http://www.kegg.jp) were analyzed as previous study (Hui et al., 2018).

### Coexpression Analysis

As a *cis*-regulator, lncRNA could regulate the expression of adjacent genes. The coexpression network of the candidate lncRNAs and their upstream or downstream 10 kb mRNAs was constructed. The connectivity and enrichment were performed due to Position Frequency Matrix.

### CeRNA Network Analysis

According to the CeRNA theory, a lncRNA-miRNA-mRNA network was constructed. The predictedinteractions of miRNA-mRNA and miRNA-lncRNA were analyzed by RNAhybrid (https://bibiserv.cebitec.uni-bielefeld.de/rnahybrid) and TargetScan (http://www.targetscan.org/).

### Quantitative Real-Time PCR (qRT-PCR)

Total RNA were extracted using Trizol reagent (TaKaRa, Dalian, China), and reverse transcription was performed by HiScript R II One Step RT-PCR kit (Vazyme, Nanjing, China). qRT-PCR was performed with ChamQ SYBR qPCR Master Mix (Vazyme, Nanjing, China) with the internal control of *β-actin* using 2^-ΔΔCt^ method. All primers sequences were showed in **Table S1**.

## Results

### Comparison of Meat Quality

**Figure 1:**
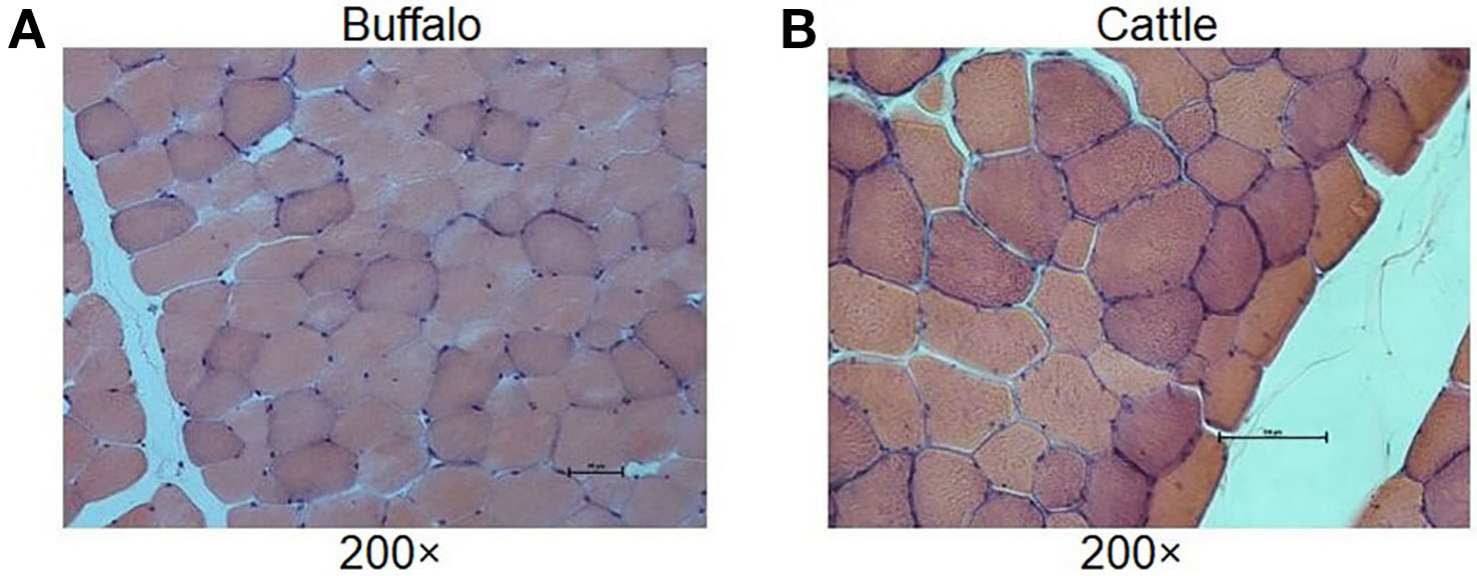
Histological analysis of longissimus dorsi muscles. The HE staining of muscle results showed that the muscle fiber area, isometric diameter, circumference and density of buffalo **(A)** were significantly smaller than those of cattle **(B)**.

In order to understand the difference of meat quality between buffalo and cattle, we analyzed the physiological and biochemical indexes of their longissimus dorsi samples. According to the indexes, significant differences were found in shear force between buffalo and cattle (*P* < 0.05), while no significant differences were found in pH, water content, intramuscular fat content and ash content (*P* > 0.05; **Table 1**). Moreover, the muscle samples were made into frozen sections and observed with HE staining by microscopy, and showed that the muscle fiber area, isometric diameter, circumference and density of buffalo were significantly smaller than those of cattle (*P* < 0.05; **Table 2** and **Figure 1**). Furthermore, the amino acid composition of muscle samples was also analyzed, and showed no difference in the ratio of essential amino acids or umami amino acids (*P* > 0.05), suggesting that under the same feeding and management conditions, the longest dorsal muscle of buffalo and cattle are basically similar in amino acid composition (**Table 3**). These results revealed that under the same feeding and management, the muscle quality of buffalo can be equivalent to that of cattle, but there are significant differences in shearing force and muscle fiber structure. Therefore, whole transcriptome RNA-Seq was performed to analyze the differences of buffalo and cattle musculus longissimus.

**Table 1:** Phenotypic parameters measured in longissimus dorsi samples.

	Buffalo (n = 3)	Cattle (n = 3)
pH	6.54 ± 0.17	6.5 ± 0.21
Shear force (N/cm)	32.90 ± 1.16	28.22 ± 0.76*
Water content (%)	77.34 ± 1.87	76.26 ± 2.67
Intramuscular fat content (%)	2.15 ± 0.91	1.84 ± 0.62
Ash (g/100 g)	1.03 ± 0.06	1.03 ± 0.7

*Significant at P < 0.05. The two breeds were compared at the same time point.

**Table 2:** Histological analysis of longissimus dorsi muscle samples.

	Buffalo (n = 20)	Cattle (n = 20)
Muscle fiber area (μm^2^)	2947.10 ± 60.97	6325.94 ± 89.71*
Isometric diameter (μm)	60.97 ± 6.08	90.15 ± 3.15*
Circumference (μm)	211.89 ± 27.97	310.93 ± 15.01*
Density (1.0 × 10^3^/mm^2^)	6.79	3.16*

*Significant at P < 0.05. The two breeds were compared at the same time point.

**Table 3:** Amino acid analysis results of longissimus dorsi muscle sample.

Amino acid	Breeds	
	Buffalo (n = 3)	Cattle (n = 3)
Asp (g/100 g)	1.81 ± 0.02	1.78 ± 0.10
Thr (g/100 g)	0.89 ± 0.02	0.91 ± 0.02
Ser (g/100 g)	0.78 ± 0.01	0.76 ± 0.02
Glu (g/100 g)	3.18 ± 0.09	3.15 ± 0.11
Pro (g/100 g)	0.66 ± 0.10	0.69 ± 0.05
Gly (g/100 g)	0.83 ± 0.04	0.85 ± 0.05
Ala (g/100 g)	1.12 ± 0.05	1.15 ± 0.03
Cys (g/100 g)	0.16 ± 0.02	0.02 ± 0.02
Val (g/100 g)	0.91 ± 0.12	0.87 ± 0.11
Met (g/100 g)	0.52 ± 0.05	0.51 ± 0.03
Ile (g/100 g)	0.93 ± 0.04	0.97 ± 0.03
Leu (g/100 g)	1.64 ± 0.06	1.64 ± 0.05
Tyr (g/100 g)	0.67 ± 0.06	0.70 ± 0.03
Phe (g/100 g)	1.00 ± 0.10	0.93 ± 0.02
Lys (g/100 g)	1.87 ± 0.07	1.80 ± 0.07
Trp (g/100 g)	0.30 ± 0.01	0.30 ± 0.02
His (g/100 g)	0.74 ± 0.12	0.72 ± 0.04
Arg (g/100 g)	1.29 ± 0.04	1.26 ± 0.06
Total amino acid content (/100 g)	19.29 ± 0.56	19.00 ± 0.51
Essential amino acid (/100 g)	8.06 ± 0.25	7.91 ± 0.10
Percentage of essential amino acids (%)	41.78 ± 0.73	41.63 ± 0.79
Flavor amino acid (/100 g)	6.94 ± 0.17	6.93 ± 0.28
Percentage of flavor amino acid (%)	35.98 ± 0.62	36.47 ± 0.33

### Ribo-Zero RNA-Seq of Buffalo and Cattle Muscle

**Figure 2:**
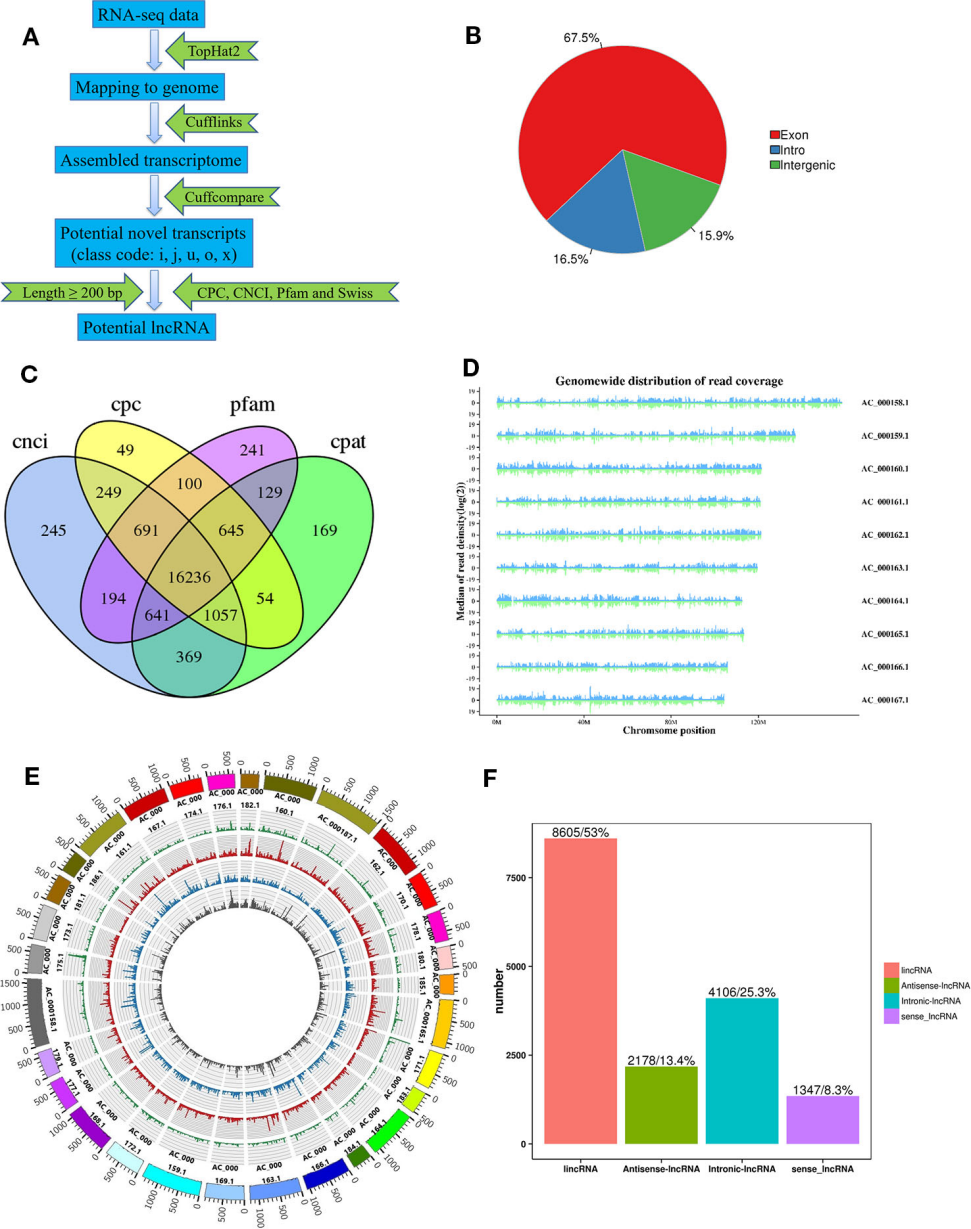
Identification of lncRNAs in buffalo and cattle skeletal muscle tissue. **(A)** Workflow for the preparation and analysis of lncRNA libraries. **(B)** Pie charts representing the percentage of reads mapping to indicated genomic regions. **(C)** Venn diagram depicting the overlap of lncRNAs discovered in lncRNAs identification. **(D**, **E)** Distribution of lncRNAs along each chromosome. **(F)** Classification of lncRNAs, as defined by their genomic location relative to neighboring or overlapping genes.

Three longissimus muscle samples were selected to perform Ribo-Zero RNA-Seq from cattle and buffalo at 12 M old, respectively. As shown in **Figure 2A**, a large number of lncRNAs were identified. On average, 83~137 and 116~131 million unique mapped clean reads were acquired from the buffalo and cattle libraries, respectively (**Table 4**). We found 67.5% of the reads located in exon regions, while a significant reduction was observed in intergenic or intronic regions (32.5%; **Figure 2B**). Novel reliable lncRNAs were filtered by using Pfam and Cpat database and tested by CPC and CNCI, and a total of 16,236 potential lncRNA transcripts were identified to be expressed (**Figure 2C**, **Table S2**). We found chromosome with longer length to be more likely to produce more lncRNAs, indicating that the number of reads distributed in the chromosome increased with chromosome length (**Figure 2D, E**). According to the Cuffcompare classes, the lncRNAs aligned to intergenic regions (u) accounted for the largest proportion (8,605, 53%; **Figure 2F**).

**Table 4:** Summary of reads mapping to the reference genome.

Samples	Total Reads	Mapped Reads	Uniq Mapped Reads	Multiple Mapped Reads	Reads Map to ‘+’	Reads Map to ‘-’
**Cattle1**	159,445,302	144,123,296 (90.39%)	116,097,555 (72.81%)	28,025,741 (17.58%)	70,917,781 (44.48%)	70,516,292 (44.23%)
**Cattle2**	182,448,004	164,895,797 (90.38%)	131,073,274 (71.84%)	33,822,523 (18.54%)	80,640,303 (44.20%)	80,556,898 (44.15%)
**Cattle3**	177,076,476	158,593,439 (89.56%)	125,617,731 (70.94%)	32,975,708 (18.62%)	77,438,780 (43.73%)	76,997,388 (43.48%)
**Buffalo1**	212,213,766	155,061,510 (73.07%)	137,479,465 (64.78%)	17,582,045 (8.29%)	77,107,211 (36.33%)	77,486,901 (36.51%)
**Buffalo2**	190,931,794	139,035,249 (72.82%)	124,008,574 (64.95%)	15,026,675 (7.87%)	69,092,223 (36.19%)	69,540,702 (36.42%)
**Buffalo3**	136,416,330	95,407,829 (69.94%)	83,824,709 (61.45%)	11,583,120 (8.49%)	47,254,024 (34.64%)	47,493,449 (34.82%)

### Genomic Features of Identified LncRNAs

**Figure 3:**
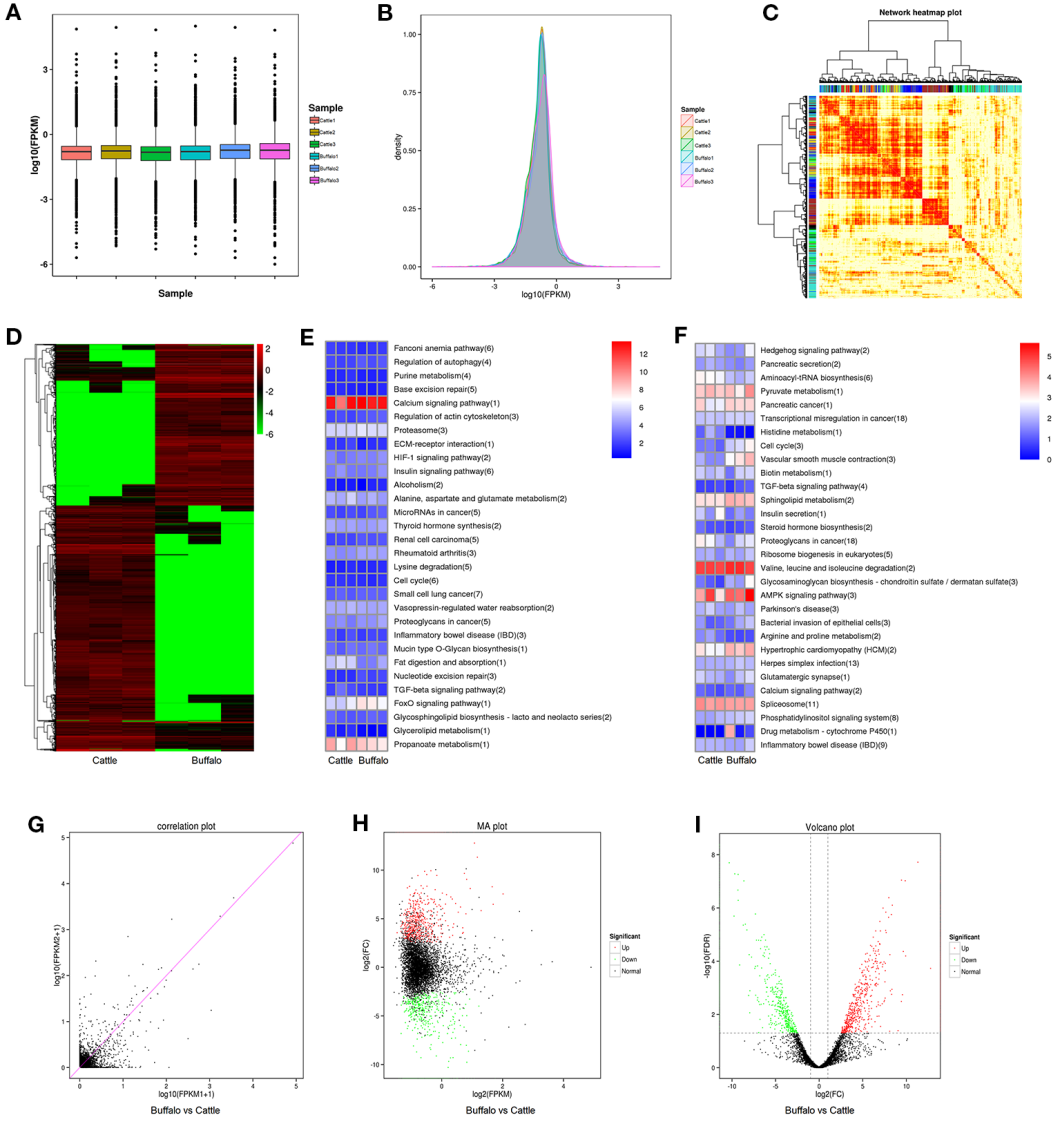
Differentially expressed lncRNAs in buffalo and cattle skeletal muscle. **(A**, **B)** The expression levels of lncRNAs, plotted as fragments per kilobase of exon per million fragments mapped (FPKM). **(C)** Weighted gene coexpression network analysis of lncRNAs in buffalo and cattle muscle sample. **(D)** Heatmap of differentially expressed lncRNAs in buffalo and cattle muscle tissue. **(E**, **F)** The top 30 enriched KEGG pathways by *cis* **(E)** and *trans* **(F)** regulation. **(G–I)** Scatter plot, MA interactive maps and volcano plot showing the correlation between abundances of individual lncRNAs in buffalo and cattle muscle sample.

As shown in **Figure 3A, B**, the identified lncRNAs showed a low expression level, and the mean expression levels were 5.96 (FPKM). As illustrated in **Figure 3C**, the lncRNA data showed a good correlation between buffalo and cattle muscle samples. 2,161 lncRNAs were significantly (*P* < 0.05) differently expressed in buffalo and cattle muscle samples (**Figure 3D**), and were listed in **Table S3**. Among all the differentially expressed lncRNAs, MSTRG.233222.1 and MSTRG.104517.1 showed the highest expression level of all up-regulated and down-regulated lncRNAs when comparing buffalo to cattle muscle tissue, respectively. To better understand potential functions of lncRNA, the scatter plot and volcano plot were displayed in **Figures 3G–I**. There were 865 lncRNAs were up-regulated, while 1,296 lncRNAs were down-regulated (*P* < 0.05; **Table S3**), and buffalo had a clear tendency for low expression of lncRNA (**Figures 3D–I**).

LncRNA regulates the transcription of coding genes through *cis* and *trans* regulatory relationships: if the role of lncRNA is limited to the same chromosome (adjacent genes), *cis* regulation is exercised; *trans* works when it affects gene expression on other chromosomes (at long distances). The top 30 enriched KEGG pathways by *cis* and *trans* regulation were present in **Figures 3E, F**. Calcium signaling pathway and Valine, leucine and isoleucine degradation had the highest level in *cis* or *trans*’ target genes cluster, respectively, indicating that these pathways may involve in regulating skeletal myogenesis.

**Figure 4:**
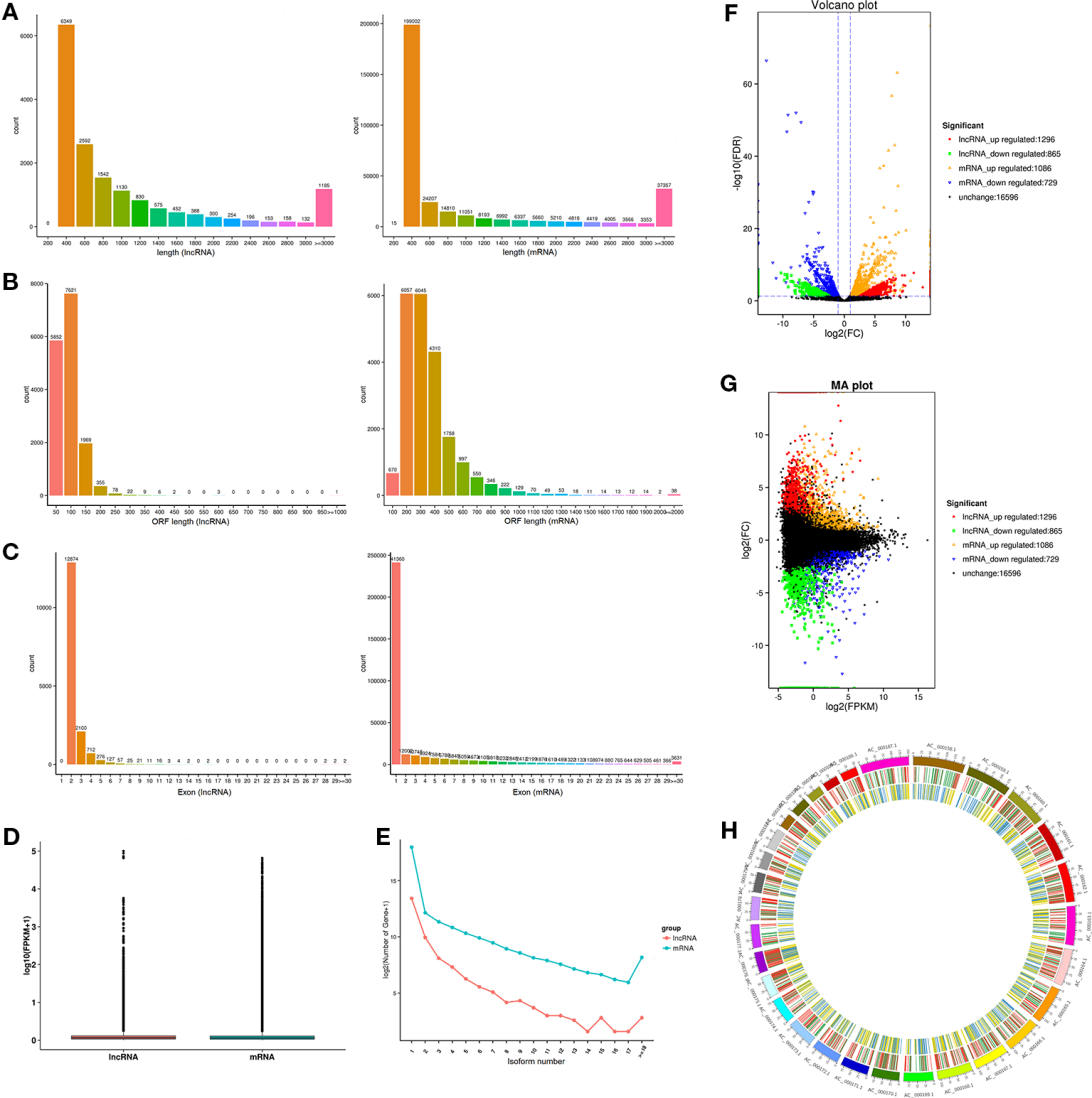
Comparison and analysis of genomic features of mRNA and lncRNA in muscle tissue. **(A)** Distribution of transcript lengths of lncRNAs and mRNA. **(B)** Distribution of ORF lengths of lncRNAs and mRNA. **(C)** Distribution of exon number of lncRNAs and mRNA. **(D)** Distribution of expression levels of lncRNAs and mRNA. **(E)** Distribution of isoform number of lncRNAs and mRNA. **(F**, **G)** Volcano plot and MA interactive maps showing the correlation between abundances of differentially expressed lncRNAs and mRNA. **(H)** Distribution of differentially expressed lncRNAs along each chromosome.

Previous study has shown that lncRNA is shorter in length than protein-encoded transcripts. As illustrated in **Figure 4A**, the mean length of lncRNAs (1,087 nucleotides) was shorter than that of the mRNA (1,153 nucleotides). The average ORF length of lncRNA was 66.7 nt, and the mRNA was 309.7 nt, revealing that lncRNA has a lower coding potential than protein-coding genes (**Figure 4B**). Moreover, lncRNAs had fewer exons (about 2.4) than protein-coding genes (about 3.6, **Figure 4C**). Interestingly, the average expression level of lncRNA was approximately 2.5-fold higher than that of protein-coding genes (6.0 vs 2.4; **Figure 4D**), revealing that lncRNAs in muscle do not act as transcriptional noise and may play important roles in regulating biological processes. Similar to mRNA, lncRNA has a similar number of isoforms, suggesting its important roles in transcriptional regulation (**Figure 4E**). The expression of lncRNA and mRNA in different combinations of comparative analysis indirectly shows the expression relationship between lncRNA and mRNA in a certain biological period, thus volcano and MA interactive maps of differentially expressed lncRNA and mRNA were drawn (**Figures 4F, G**). The positional relationship of genes on chromosomes is closely related to the functions of genes, and some lncRNAs may have regulatory functions on their neighboring genes. Therefore, we analyzed the chromosome distribution of differentially expressed lncRNAs and mRNAs (**Figure 4H**).

### Coexpression and CeRNAs Networks

**Figure 5:**
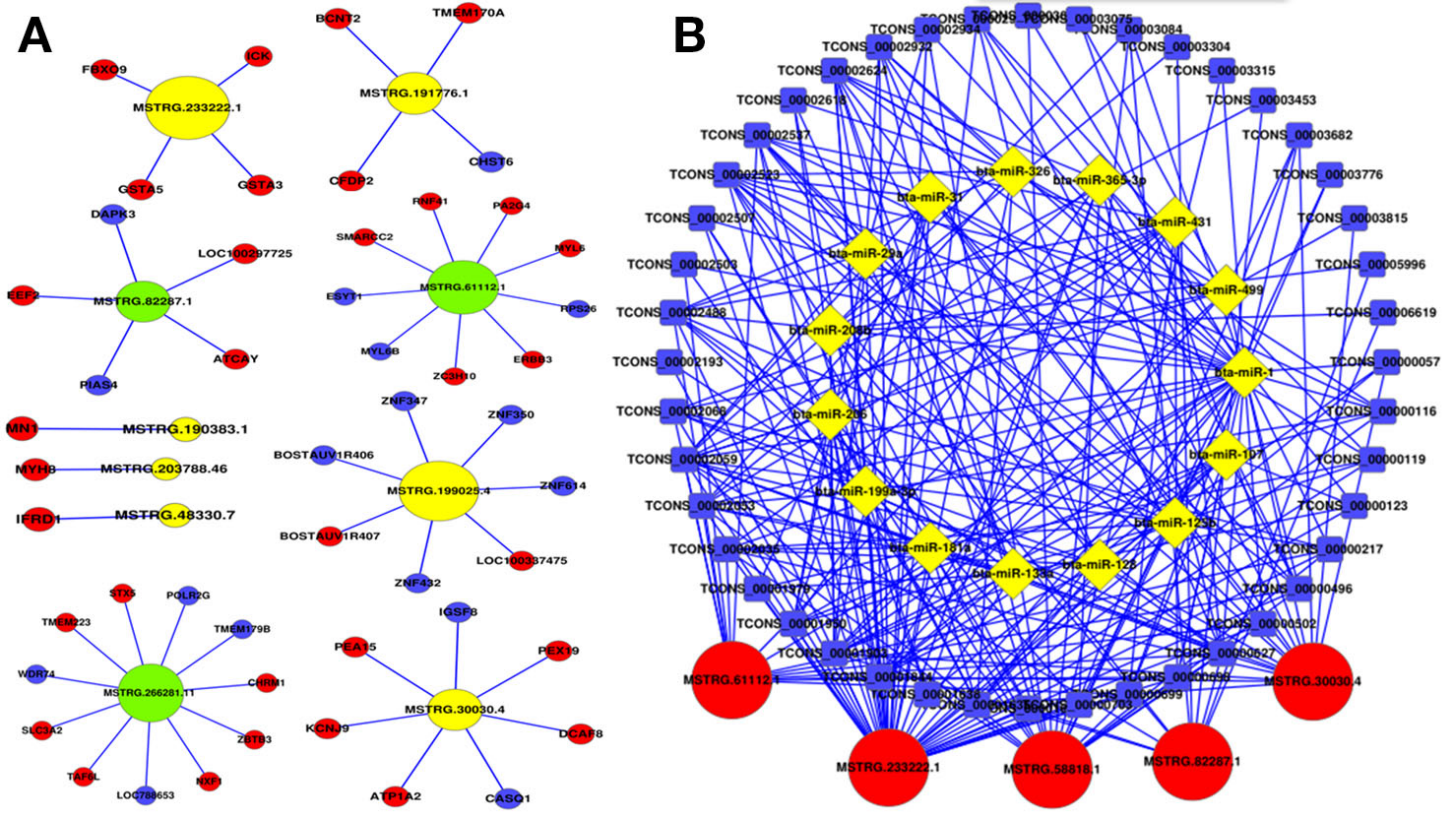
Coexpression network and competing endogenous RNA network in cattle and buffalo muscle tissues. **(A)** LncRNAs and their potential *cis* regulated nearby genes are shown in the network. **(B)** The network includes lncRNA-miRNA and miRNA-mRNA interactions, whereby edges indicate sequence matching, and lncRNAs connect ties suggesting miRNA-mediated mRNA expression.

In order to further investigate the *cis*-regulatory relationship of lncRNAs, the adjacent coding genes 10 kb upstream and downstream of the candidate lncRNAs were performed to construct coexpression network. The top 10 most significantly differentially expressed lncRNAs were chosen to hunt their neighboring coding genes (**Figure 5A**). Each lncRNA had different number of adjacent coding genes. For example, MSTRG.266281.11 had maximum number of 11 neighboring coding genes, whereas MSTRG.203788.46 had only one nearby coding gene (MYH8) and was positively correlated with expression levels of MYH8. Interestingly, MSTRG.233222.1 were up-regulated in the buffalo muscle compared to the cattle muscle, and all its four neighboring coding genes (ICK, GSTA5, FBXO9, GSTA3) presented higher levels in the buffalo muscle, suggesting this lncRNA may has *cis*-regulatory relationship on its neighboring genes. The coexpression network could furnish valuable clue for these lncRNAs’ potential function in regulating nearby coding genes.

LncRNAs to sequester miRNAs from their target mRNAs could be as a member of ceRNAs, and miRNAs act as common target of the lncRNAs and mRNAs. 15 muscle development related miRNAs were chosen with a total of 5 lncRNAs and 44 mRNAs to construct an ceRNA (mRNA-miRNA-lncRNA) network (**Figure 5B**). For instance, MSTRG.30030.4 has multiple binding sites for muscle-related microRNAs, for example, miR-133a and miR-128 (Chen et al., 2006; Kim et al., 2006). This ceRNA network may provide valuable information for buffalo skeletal myogenesis.

### Identification of Candidate LncRNA

**Figure 6:**
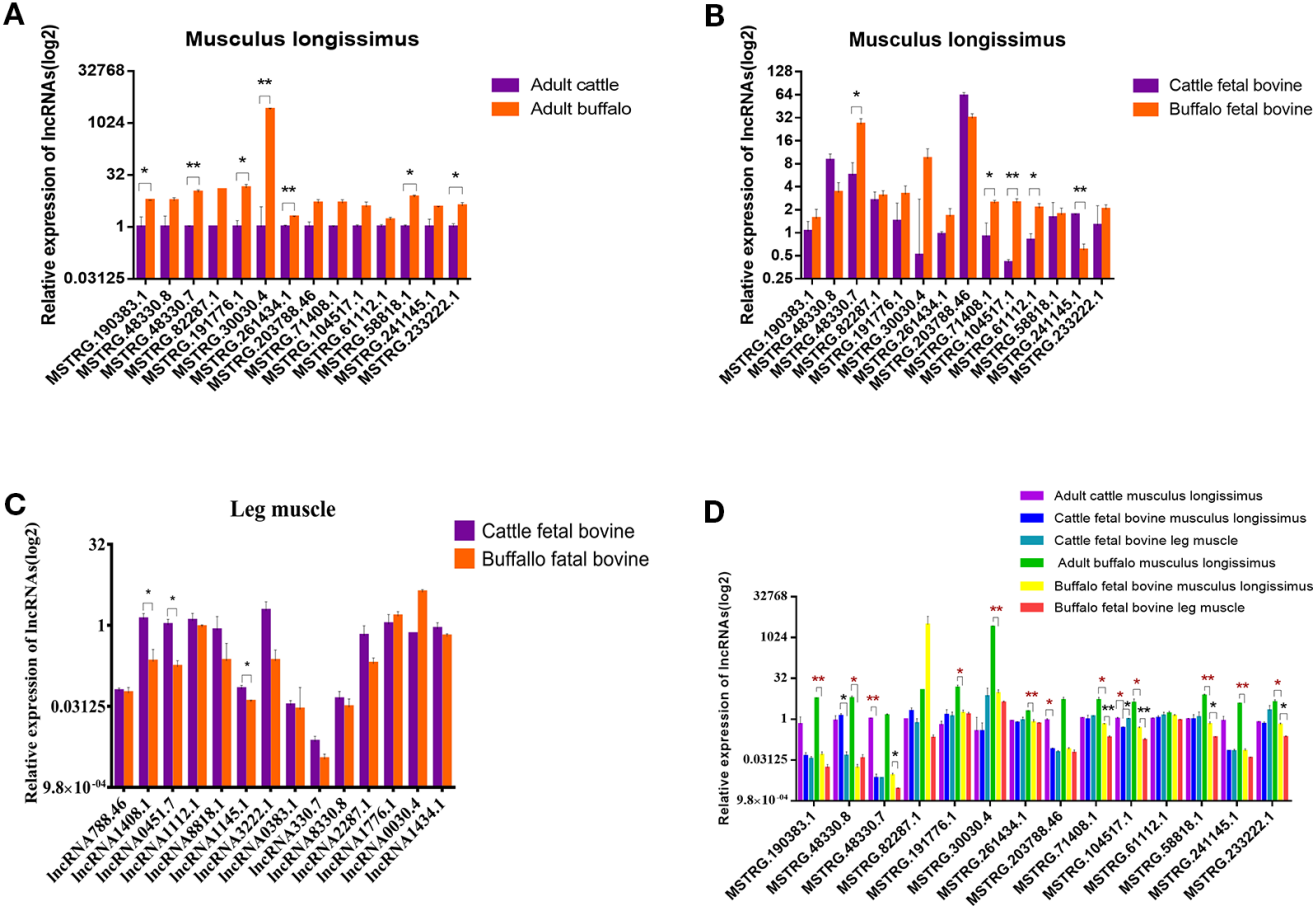
Validation of putative lncRNA. **(A)** 14 lncRNAs were selected and identified, as they exhibited significantly different expression patterns (assessed from our RNA-sequencing approach) when comparing longissimus dorsi muscles, using quantitative real-time PCR (qRT-PCR). **(B)** 14 lncRNAs were identified in fetus cattle and buffalo longissimus dorsi muscles using qRT-PCR. **(C)** 14 lncRNAs were identified in fetus cattle and buffalo leg muscles using qRT-PCR. **(D)** The expression of 14 lncRNAs in cattle and buffalo muscle tissue. *P < 0.05, **P < 0.01.

To further validate lncRNAs expression profiles obtained from the RNA-Seq results, 14 lncRNAs that may be involved in muscle development regulation were selected and measured by qRT-PCR. The normalized read counts of the 14 lncRNAs were shown in **Table S2**. Overall, these randomly selected lncRNAs showed similar expression patterns between qRT-PCR and sequencing results, suggesting that the lncRNA-Seq data are highly accurate (**Figure 6A**). Similarly, we also analyzed the expression of these 14 lncRNAs in the fetal dorsal longest muscle and found that the expression of these 14 lncRNAs varied more in the fetal period, indicating that muscle development was more complex in the fetal period and lncRNA was involved in this process (**Figure 6B**). We also analyzed the changes of lncRNA in fetal leg muscles and found that the change trend was basically consistent with the expression of lncRNA in the longest dorsal muscle (**Figure 6C**) As shown in **Figure 6D**, MSTRG.30030.4 and MSTRG.104517.1 were the lncRNAs with the most significant expression differences among the highly expressed lncRNAs, revealing that they may play important roles in muscle growth or differentiation. Therefore, we also examined the expression of 14 lncRNAs in different tissues in order to find potential lncRNAs that are specifically expressed and highly expressed in muscles.

**Figure 7:**
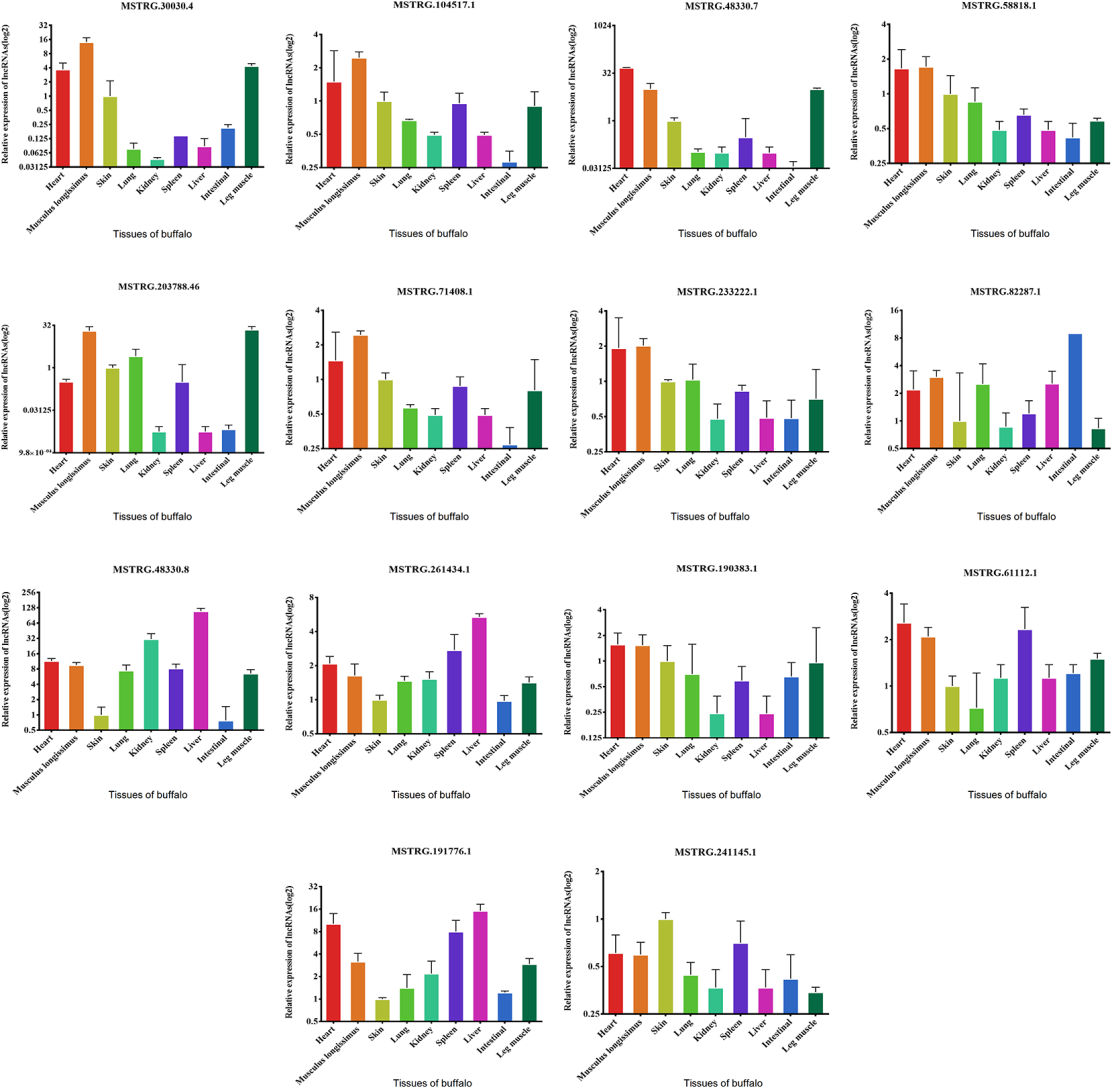
Expression levels of 14 candidate lncRNAs in different tissues of fetus buffalo.

**Figure 8:**
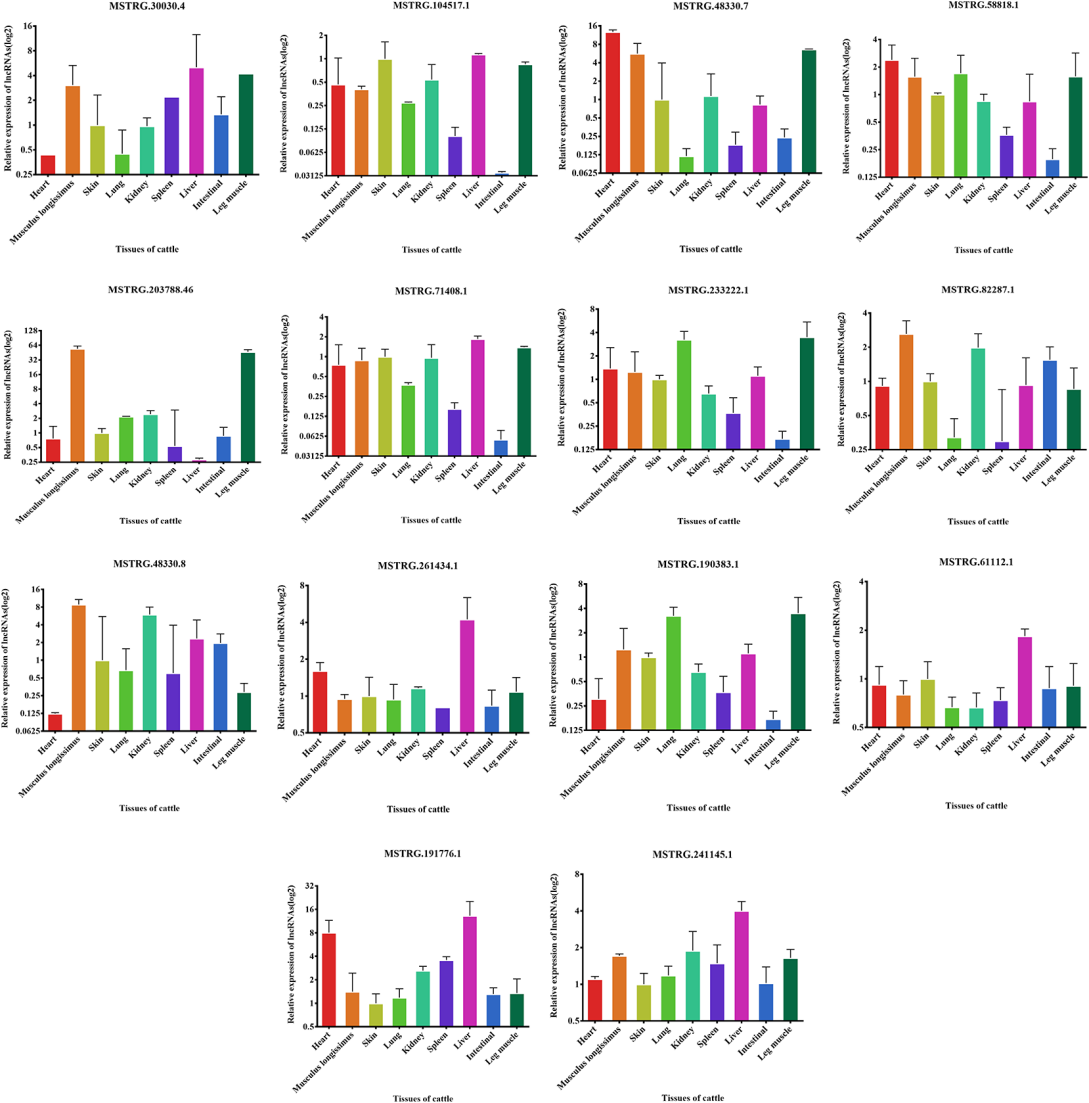
Expression levels of 14 candidate lncRNAs in different tissues of fetus cattle.

We examined the expression of these 14 lncRNAs in the heart, spleen, lung, liver, kidney, skin, small intestine, brain, leg muscle and dorsal longest muscle of cattle and buffalo. The buffalo tissue expression assay displayed that MSTRG.30030.4, MSTRG.104517.1, MSTRG.48330.7, MSTRG.58818.1, MSTRG.71408.1, MSTRG.203788.46, and MSTRG.233222.1 was highly expressed in muscle tissue and low in other tissues, indicating potential roles in buffalo muscle development (**Figure 7**). Similarly, we found that MSTRG.48330.7, MSTRG.30030.4, and MSTRG.203788.46 were highly and specifically expressed in cattle muscle tissue, and these lncRNAs could be chosen as candidates to analyze their real roles *in vivo* and *in vitro* in muscle development (**Figure 8**).

## Discussion

Muscle strength is a quantitative trait, which is related to a variety of physiological and biochemical indexes. The origin and evolution of buffalo are closely related to the cultivation of human beings (Low et al., 2019). Due to the rise of industrial revolution and the improvement of social productivity, few buffaloes are still retained for farming. At present, buffaloes were selected for meat or milk production, and their easement value is gradually abandoned (Pisano et al., 2016). By analyzing the longest dorsal muscle of buffalo and cattle in the same breeding and growing environment, it was found that their meat value was comparable. Therefore, high quality buffalo meat can be obtained through optimization of breeding management. Muscle freshness is related to the composition and content of umami amino acids in muscle. The flavor of beef is related to fatty acid content, and marbling level of beef is one of the important indicators in beef classification. Present study, focus on physiological and biochemical indexes, amino acid composition, and intramuscular fat content of the longest dorsal muscle of boar buffalo and local cattle were not significantly different under the same feeding and management conditions. Interestingly, there are significant differences in the shear force and muscle fiber structure (muscle fiber area, diameter, and circumference) between buffalo and cattle, which may be due to genetic factors rather than the influence of breeding management on this trait. These results suggest that the strength trait of buffalo is positively selected, which is related to the role of buffalo in providing animal power under China's small-scale peasant economy for thousands of years. Similarly, candidate genes associated with strength trait were also positively selected.

Most of the studies on the molecular mechanism of skeletal muscle development in bovine are protein coding genes. However, the occurrence and potential functions of lncRNAs, which reflect the differences between the longissimus muscles of buffalo and cattle, are still unclear. Abundant lncRNAs were differentially expressed in the muscle tissue of buffalo and cattle, suggesting that lncRNAs have specific roles in muscle but not by-product of mRNA. In addition, some lncRNAs were specifically or mainly expressed in muscle tissue, such as MSTRG.30030.4, revealing that these lncRNAs are purposefully produced. LncRNA is more than just a by-product of protein coding genes, and many lncRNAs have been demonstrated to play a role in skeletal muscle development. Increasing studies show that lncRNA *in cis* or *in trans* is involved in the transcriptional or post-transcriptional regulation of gene expression (Caretti et al., 2006; Korostowski et al., 2012; Han et al., 2014; Zhou et al., 2015).

The top 10 most significantly differentially expressed lncRNAs were chosen with their neighboring coding genes to construct a co-express network. For example, MSTRG.233222.1 were up-regulated in the buffalo muscle, and all its four neighboring coding genes presented higher levels in the buffalo muscle, suggesting this lncRNA may has *cis*-regulatory relationship on its neighboring genes. AmRNA-miRNA-lncRNA network was constructed in buffalo muscle according to the common target miRNAs of the mRNAs and lncRNAs. MSTRG.30030.4 has multiple binding sites for muscle-related microRNAs, for example, miR-133a and miR-128. Tissue expression analysis showed that lncRNA MSTRG.48330.7, MSTRG.30030.4, and MSTRG.203788.46 were mainly expressed in muscle tissue, that revealing its potential role in buffalo muscle development. Moreover, MSTRG.48330.7, MSTRG.30030.4, and MSTRG.203788.46 had multiple binding sites for muscle-related microRNAs, for example, miR-1/206 and miR-133a which were the most representative muscle-associated miRNAs (Chen et al., 2006; Kim et al., 2006). Therefore, our next step is to explore the role of MSTRG.48330.7, MSTRG.30030.4, and MSTRG.203788.46 in the differentiation of cattle and buffalo myoblasts.

## Conclusions

This study is the first to compare chemical-physical characteristics of muscle in cattle and buffalo, and provide an overview of lncRNA expression in buffalo muscle tissues. Thousands of lncRNAs were identified, and several of which were highly and specifically expressed in buffalo muscle tissues. We further constructed coexpression and ceRNA networks, and found MSTRG.48330.7, MSTRG.30030.4, and MSTRG.203788.46 could be as ceRNA which contained potential binding sites for miR-1/206 and miR-133a. This study may lay a foundation for in-depth investigations into the roles of those lncRNAs during buffalo muscle development.

## Data Availability Statement

The data generated and transcripts obtained were deposited at NCBI as the SRA accession SRP116252 and TSA accession GFWP00000000.1. The data and material are also provided as **Supplementary Data**.

## Ethics Statement

All experimental animals were provided by SIYE Breeding Farm, Nanning, Guangxi Province, China. The cattles and buffalos were maintained according to the No. 5 Proclamation of the Ministry of Agriculture, P. R. China, and we confirm that all animal protocols and methods were approved by the Review Committee for the Use of Animal Subjects of Guangxi University.

## Author Contributions

QL, JR, and HL designed the experiment. KH and PW collected the experimental tissues. KH, PW, TF, and KC contributed to analyzing the data and interpreting the results. HL, QL, and QQ wrote the manuscript with input from all the authors. DS, CL, and LS participated in designing the structure of the article. All the authors read and approved the final manuscript.

## Funding

This work was supported by the National Natural Science Foundation of China (Grant No. 31860638 and 31760648) and the Guangxi Natural Science Foundation (Grant No. AA17204051, 2018GXNSFAA050086 and AB16380042).

## Conflict of Interest

The authors declare that the research was conducted in the absence of any commercial or financial relationships that could be construed as a potential conflict of interest.

## Abbreviations

CeRNA, competing endogenous RNA; miRNA, microRNA; lncRNA, long non-coding RNA; qRT-PCR, real-time quantitative PCR.

## References

[B1] Anderson D. M. Anderson K. M. Chang C. L. Makarewich C. A. Nelson B. R. McAnally J. R. (2015). A micropeptide encoded by a putative long noncoding RNA regulates muscle performance. Cell 160, 595–606. 10.1016/j.cell.2015.01.009 25640239 PMC4356254

[B2] Buckingham M. Relaix F. (2015). PAX3 and PAX7 as upstream regulators of myogenesis. Semin. Cell Dev. Biol. 44, 115–125. 10.1016/j.semcdb.2015.09.017 26424495

[B3] Caretti G. Schiltz R. L. Dilworth F. J. Padova M. D. Zhao P. Ogryzko V. (2006). The RNA helicases p68/p72 and the noncoding RNA SRA are coregulators of MyoD and skeletal muscle differentiation. Dev. Cell 11, 547–560. 10.1016/j.devcel.2006.08.003 17011493

[B4] Cesana M. Cacchiarelli D. Legnini I. Santini T. Sthandier O. Chinappi M. (2011). A long noncoding RNA controls muscle differentiation by functioning as a competing endogenous RNA. Cell 147, 358–369. 10.1016/j.cell.2011.09.028 22000014 PMC3234495

[B5] Chen J. F. Mandel E. M. Thomson J. M. Wu Q. Callis T. E. Hammond S. M. (2006). The role of microRNA-1 and microRNA-133 in skeletal muscle proliferation and differentiation. Nat. Genet. 38, 228–233. 10.1038/ng1725 16380711 PMC2538576

[B6] Han P. Li W. Lin C. H. Yang J. Shang C. Nurnberg S. T. (2014). A long noncoding RNA protects the heart from pathological hypertrophy. Nature 514, 102–106. 10.1038/nature13596 25119045 PMC4184960

[B7] Hernandez-Hernandez J. M. Garcia-Gonzalez E. G. Brun C. E. Rudnicki M. A. (2017). The myogenic regulatory factors, determinants of muscle development, cell identity and regeneration. Semin. Cell Dev. Biol. 72, 10–18. 10.1016/j.semcdb.2017.11.010 29127045 PMC5723221

[B8] Hui L. Jiameng Y. Rui J. Xuefeng W. Chengchuang S. Yongzhen H. (2018). Long Non-coding RNA profiling reveals an abundant MDNCR that promotes differentiation of myoblasts by sponging miR-133a. Mol. Ther. Nucleic Acids 12, 610–625. 10.1016/j.omtn.2018.07.003 30195797 PMC6078111

[B9] Kallen A. N. Zhou X. B. Xu J. Qiao C. Ma J. Yan L. (2013). The imprinted H19 lncRNA antagonizes let-7 microRNAs. Mol. Cell 52, 101–112. 10.1016/j.molcel.2013.08.027 24055342 PMC3843377

[B10] Kim H. K. Lee Y. S. Sivaprasad U. Malhotra A. Dutta A. (2006). Muscle-specific microRNA miR-206 promotes muscle differentiation. J. Cell Biol. 174, 677–687. 10.1083/jcb.200603008 16923828 PMC2064311

[B11] Korostowski L. Sedlak N. Engel N. (2012). The Kcnq1ot1 long non-coding RNA affects chromatin conformation and expression of Kcnq1, but does not regulate its imprinting in the developing heart. PLoS Genet. 8, e1002956. 10.1371/journal.pgen.1002956 23028363 PMC3447949

[B12] Li H. Yang J. Jiang R. Wei X. Song C. Huang Y. (2018a). CircFUT10 reduces proliferation and facilitates differentiation of myoblasts by sponging miR-133a. J. Cell. Physiol. 233, 4643–4651. 10.1002/jcp.26230 29044517

[B13] Li Y. Chen X. Sun H. Wang H. (2018b). Long non-coding RNAs in the regulation of skeletal myogenesis and muscle diseases. Cancer Lett. 417, 58–64. 10.1016/j.canlet.2017.12.015 29253523

[B14] Low W. Y. Tearle R. Bickhart D. M. Rosen B. D. Kingan S. B. Swale T. (2019). Chromosome-level assembly of the water buffalo genome surpasses human and goat genomes in sequence contiguity. Nat. Commun. 10, 260. 10.1038/s41467-018-08260-0 30651564 PMC6335429

[B15] Mchugh C. A. Chen C. K. Chow A. Surka C. F. Tran C. Mcdonel P. (2015). The Xist lncRNA interacts directly with SHARP to silence transcription through HDAC3. Nature 521, 232. 10.1038/nature14443 25915022 PMC4516396

[B16] Munschauer M. Nguyen C. T. Sirokman K. Hartigan C. R. Hogstrom L. Engreitz J. M. (2018). The NORAD lncRNA assembles a topoisomerase complex critical for genome stability. Nature 561, 132. 10.1038/s41586-018-0453-z 30150775

[B17] Nelson B. R. Makarewich C. A. Anderson D. M. Winders B. R. Troupes C. D. Wu F. (2016). A peptide encoded by a transcript annotated as long noncoding RNA enhances SERCA activity in muscle. Science 351, 271–275. 10.1126/science.aad4076 26816378 PMC4892890

[B18] Neumann P. Nicolas Jaé Knau A. Glaser S. F. Fouani Y. Rossbach O. (2018). The lncRNA GATA6-AS epigenetically regulates endothelial gene expression *via* interaction with LOXL2. Nat. Commun. 9, 237. 10.1038/s41467-017-02431-1 29339785 PMC5770451

[B19] Pisano M. B. Scano P. Murgia A. Cosentino S. Caboni P. (2016). Metabolomics and microbiological profile of Italian mozzarella cheese produced with buffalo and cow milk. Food Chem. 192, 618–624. 10.1016/j.foodchem.2015.07.061 26304391

[B20] Sakaridis I. Ganopoulos I. Argiriou A. Tsaftaris A. (2013). A fast and accurate method for controlling the correct labeling of products containing buffalo meat using High Resolution Melting (HRM) analysis. Meat Sci. 94, 84–88. 10.1016/j.meatsci.2012.12.017 23403299

[B21] Wang J. Gong C. Maquat L. E. (2013). Control of myogenesis by rodent SINE-containing lncRNAs. Genes Dev. 27, 793–804. 10.1101/gad.212639.112 23558772 PMC3639419

[B22] Wang G. Q. Wang Y. Xiong Y. Chen X. C. Ma M. L. Cai R. (2016). Sirt1 AS lncRNA interacts with its mRNA to inhibit muscle formation by attenuating function of miR-34a. Sci. Rep. 6, 21865. 10.1038/srep21865 26902620 PMC4763196

[B23] Zhang Y. Wang H. Gui L. Wang H. Mei C. Zhang Y. (2016). Profile of muscle tissue gene expression specific to water buffalo: comparison with domestic cattle by genome array. Gene 577, 24–31. 10.1016/j.gene.2015.11.015 26598327

[B24] Zhou L. Sun K. Zhao Y. Zhang S. Wang X. Li Y. (2015). Linc-YY1 promotes myogenic differentiation and muscle regeneration through an interaction with the transcription factor YY1. Nat. Commun. 6. 10.1038/ncomms10026 26658965

