# Supplementary figures and images for: Comparison of Long Non-Coding RNA Expression Profiles of Cattle and Buffalo Differing in Muscle Characteristics
Source: Front Genet. 2020 Feb 26;11:98. doi: 10.3389/fgene.2020.00098 (PMC7054449; doi:10.3389/fgene.2020.00098)

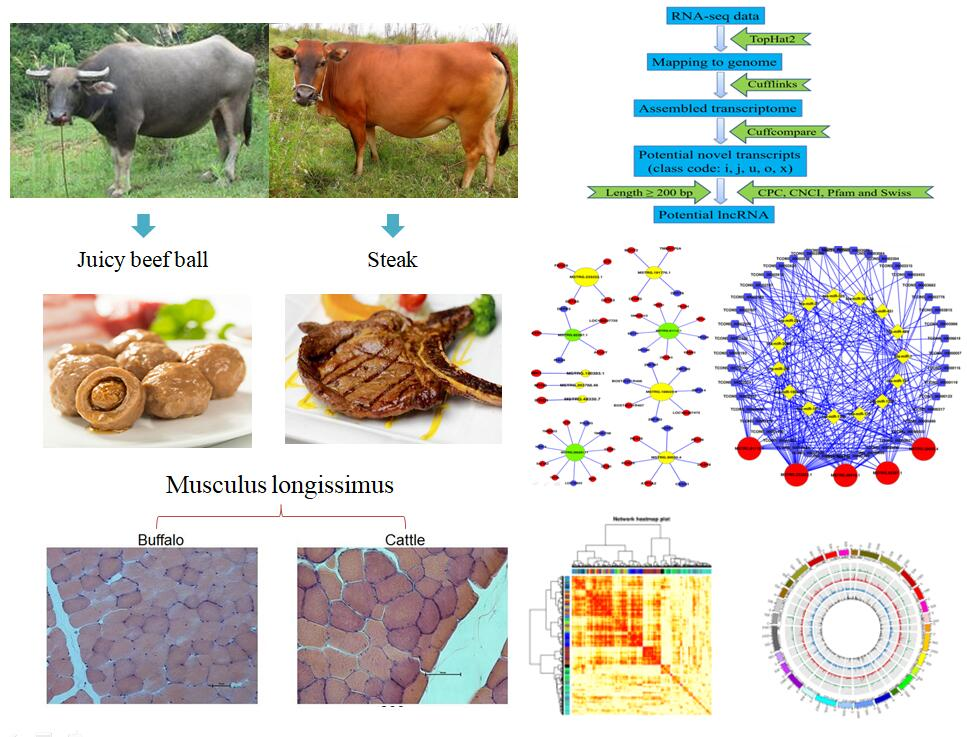

Supplement: Supplementary file 1 [file Image_1.tif]
