# Supplementary material for: Comparison of Long Non-Coding RNA Expression Profiles of Cattle and Buffalo Differing in Muscle Characteristics
Source: Front Genet. 2020 Feb 26;11:98. doi: 10.3389/fgene.2020.00098 (PMC7054449; doi:10.3389/fgene.2020.00098)
Supplement: Supplementary file 2 [file Table_1.docx]

**Table S1 primers for qPCR**

| **LncRNA** | **Forward primer 5’→3’** | **Reverse primer 5’→3’** |
| --- | --- | --- |
| MSTRG.58818.1 | TGTTGATTCGGCTGATCTGGC | TATACCCTTGACCGAAGACCG |
| MSTRG.61112.1 | AAGTGGAGGAAGAAGCGAATG | GTAGGTCTCGCAAAGGTGGTC |
| MSTRG.71408.1 | GTTGCCTAAGGAGGGGTGAAC | TACAGTGGCTATTCACAGGCG |
| MSTRG.104517.1 | CGGCATCAATATGGTGACCTC | GGCGCGATCCCACTACTG |
| MSTRG.233222.1 | CTGGCTGGCTAGGCGCTCCAT | TGGATGTGTCTGGAGTCTTGG |
| MSTRG.82287.1 | GCGTGGACAGAACTGTGGGT | CCTGCGTCTAGCCACTCTTGA |
| MSTRG.30030.4 | GTTGCGACTACTCATTCCTA | TACTGTCTGTTGCCTGTTCAT |
| MSTRG.48330.7 | ATTCAGCCTTGAGCACATC | GGTCCTCAGTTATAGACGAAAC |
| MSTRG.261434.1 | GACTCGCTGAATCGCCCAGTT | GCTCCTCGGGACTCATCCAAG |
| MSTRG.191776.1 | ATGCTGGAACTTGTAGTGCTCAGA | GCGAATAACCCTGTGAATAGCGA |
| MSTRG.241145.1 | TGCCATCCAGCCATCTCATCCT | GCCACCTCATGCGAAGAGTTGA |
| MSTRG.190383.1 | CCGAACAGCAGAGGACAGAAGC | GAAACCGAGGGTTGGTGGAAGG |
| MSTRG.82287.1 | ACTAAGCGGGTGGTGTTAGATTGG | GGCAGATGGCATGGAAGGACAG |
| MSTRG.203788.46 | CTCTGTGGCGTGTTTCTCCTTCTC | GAGAGCCGAGGATGAGGAAGAGAT |
| MSTRG.48330.8 | TTGGCTGATATGGAACCTGACACA | CCTCTGATTGGTCTGGAGTCTTGT |
| β-actin | CTGGCATTGTCATGGACTCTG | GCTCGGCTGTGGTGGTAAA |
